# The clinical course of idiopathic pulmonary fibrosis and its association to quality of life over time: longitudinal data from the INSIGHTS-IPF registry

**DOI:** 10.1186/s12931-019-1020-3

**Published:** 2019-03-15

**Authors:** Michael Kreuter, Jeff Swigris, David Pittrow, Silke Geier, Jens Klotsche, Antje Prasse, Hubert Wirtz, Dirk Koschel, Stefan Andreas, Martin Claussen, Christian Grohé, Henrike Wilkens, Lars Hagmeyer, Dirk Skowasch, Joachim F. Meyer, Joachim Kirschner, Sven Gläser, Nicolas Kahn, Tobias Welte, Claus Neurohr, Martin Schwaiblmair, Matthias Held, Thomas Bahmer, Tim Oqueka, Marion Frankenberger, Jürgen Behr

**Affiliations:** 1 Center for interstitial and rare lung diseases, Thoraxklinik, University of Heidelberg, Röntgenstr 1, D-69126 Heidelberg, Germany; 2 Interstitial Lung Disease Program, National Jewish Health, Denver, CO USA; 3 Institut für Klinische Pharmakologie, Medizinische Fakultät, Technische Universität Dresden, Dresden, Germany; 4 Department Market Access, Boehringer Ingelheim, Ingelheim am Rhein, Germany; 5 Epidemiologie, Deutsches Rheuma-Forschungsinstitut, Berlin, Germany; 6 Klinik für Pneumologie, Medizinische Hochschule Hannover, Hannover, Germany; 7 Fraunhofer Institute ITEM, Hannover, Germany; 8 Abteilung für Pneumologie, Department Innere Medizin, Neurologie und Dermatologie, Universitätsklinikum Leipzig AöR, Leipzig, Germany; 9 Zentrum für Pneumologie und Bereich Pneumologie, Fachkrankenhaus Coswig und Uniklinikum Dresden, Coswig, Germany; 10 Lungenfachklinik Immenhausen and Universitätsmedizin Göttingen, Kardiologie und Pneumologie, Göttingen, Germany; 11 Lungen Clinic Grosshansdorf, Grosshansdorf, Germany; 12 Klinik für Pneumologie – ELK, Berlin Buch, Berlin, Germany; 13 Klinik für Innere Medizin V, Pneumologie, Universitätsklinikum Universitätskliniken des Saarlandes, Homburg, Germany; 14 Krankenhaus Bethanien, Solingen, Germany; 15 Medizinische Klinik und Poliklinik II, Universitätsklinikum Bonn, Bonn, Germany; 16 Lungenzentrum München, LZM Bogenhausen-Harlaching, Städtisches Klinikum München GmbH, Munich, Germany; 17 Center for Internal Medical Studies CIMS, Bamberg, Germany; 18 Klinik und Poliklinik für Innere Medizin B, Forschungsbereich Pneumologie und Pneumologische Epidemiologie, Universitätsmedizin Greifswald, Greifswald, Germany; 19 Vivantes Klinikum Spandau, Klinik für Innere Medizin, Berlin, Germany; 20 Medizinische Klinik und Poliklinik V, Klinikum der LMU, Munich, Germany; 21 Medizinische Klinik, Klinikum Augsburg, Augsburg, Germany; 22 Klinikum Würzburg Mitte, Standort Missioklinik, Abteilung Innere Medizin, Pneumologie, Würzburg, Germany; 23 Asklepios Fachkliniken München-Gauting, Munich, Germany; 24 Schillerhöhe, Robert Bosch Krankenhaus, Stuttgart, Germany; 25 Universitätsklinikum Hamburg, Hamburg, Germany; 26 German Center for Lung Research, gießen, Germany

**Keywords:** Idiopathic pulmonary fibrosis, Patient-related outcomes, Cohort study, SQRQ

## Abstract

**Background:**

Quality of life (QoL) is profoundly impaired in patients with idiopathic pulmonary fibrosis (IPF). However, data is limited regarding the course of QoL. We therefore analysed longitudinal data from the German INSIGHTS-IPF registry.

**Methods:**

Clinical status and QoL were assessed at enrollment and subsequently at 6- to 12-months intervals. A range of different QoL questionnaires including the St. George’s Respiratory Questionnaire (SGRQ) were used.

**Results:**

Data from 424 patients were included; 76.9% male; mean age 68.7 ± 9.1 years, mean FVC% predicted 75.9 ± 19.4, mean DL_CO_% predicted 36.1 ± 15.9. QoL worsened significantly during follow-up with higher total SGRQ scores (increased by 1.47 per year; 95% CI: 1.17 to 1.76; *p* < 0.001) and higher UCSD-SOBQ scores and lower EQ-5D VAS and WHO-5 scores. An absolute decline in FVC% predicted of > 10% was associated with a significant deterioration in SGRQ (increasing by 9.08 units; 95% CI: 2.48 to 15.67; *p* = 0.007), while patients with stable or improved FVC had no significantly change in SGRQ. Patients with a > 10% decrease of DL_CO %_ predicted also had a significant increase in SGRQ (+ 7.79 units; 95% CI: 0.85 to 14.73; *p* = 0.028), while SQRQ was almost stable in patients with stable or improved DL_CO_. Patients who died had a significant greater increase in SGRQ total scores (mean 11.8 ± 18.6) at their last follow-up visit prior to death compared to survivors (mean 4.2 ± 18.9; HR = 1.03; 95% CI: 1.01 to 1.04; *p* < 0.001). All QoL scores across the follow-up period were significantly worse in hospitalised patients compared to non-hospitalised patients, with the worst scores reported in those hospitalised for acute exacerbations.

**Conclusions:**

QoL assessments in the INSIGHTS-IPF registry demonstrate a close relationship between QoL and clinically meaningful changes in lung function, comorbidities, disease duration and clinical course of IPF, including hospitalisation and mortality.

**Electronic supplementary material:**

The online version of this article (10.1186/s12931-019-1020-3) contains supplementary material, which is available to authorized users.

## Introduction

Idiopathic pulmonary fibrosis (IPF) is a severe disease characterised by debilitating symptoms such as cough, fatigue, increasing and immobilising dyspnoea and is associated with a limited survival [1, 2]. IPF symptoms and outcomes are also strongly influenced by co-morbidities [3, 4]. The psychosocial impact of IPF is substantial for both patients and their caregivers [1, 5]. Recent reports have shown that in IPF patients health-related quality of life (QoL) is associated with clinical symptoms, duration of disease, physical activity, comorbidities, and disease severity [6–10] and may be prognostic [11].

While current therapies aim to improve the disease course and to prolong life expectancy, less well understood is how best to improve an individual’s health-related quality of life and psychosocial needs [12]. To address this, patient-reported outcomes incorporating determination of health-related quality of life (QoL) assessments are increasingly used as important outcome measures, in clinical practice and in clinical trials [6]. However, data on longitudinal determinations of QoL in IPF patients, especially in the real-life setting are sparse. Previously, we have reported on baseline characteristics and QoL status in patients enrolled in a large nationwide observational IPF registry (INSIGHTS-IPF) [2, 7]. In the present analyses, we report longitudinal data (with up to 36 months follow-up) for a range of QoL measures and examine changes in QoL outcomes and their relationship to clinical parameters.

## Methods

### Study population

The INSIGHTS-IPF (“Investigating significant health trends in idiopathic pulmonary fibrosis”) registry is a nationwide, investigator-initiated cohort study, which has been continuously enrolling consecutive patients in routine clinical care across 19 pulmonary specialist centres in Germany since November 2012. Patients ≥18 years of age with a study-site diagnosis of IPF after provision of written informed consent can be enrolled, with no explicit exclusion criteria. The registry’s structure, methodology and regulatory aspects, and a detailed description of the baseline characteristics of the patient cohort have been reported [2, 13, 14]. Patients with a QoL assessment at baseline and at least one follow-up assessment were available for this study. The change in QoL was studied for 3 years after enrolment into the registry (i.e., 3-year follow-up), because of the currently limited number of patients (*n* = 24) with available QoL data for follow-up beyond this time-point.

### Clinical and patient-reported outcome measures

Data were collected at enrolment (baseline) and at subsequent 6- to 12 months intervals. At each follow-up visit, all clinical events including hospitalisation and acute exacerbations (as judged by the treating physician), and death were recorded by each site. At these visits, a range of routine pulmonary function tests were documented, including forced vital capacity (FVC) % predicted, diffusing capacity of the lung for carbon monoxide (DL_CO_) % predicted, the forced expiratory volume in 1 s (FEV_1_), and six-minute walk distance (6MWD). The Gender, Age, Physiology (GAP) index was also determined. All patients completed the NYHA functional status and a range of QoL questionnaires as previously reported [7]. These include the St. George’s Respiratory Questionnaire (SGRQ), the University of California San Diego Shortness of Breath Questionnaire (USCD-SOBQ), the EuroQol five-dimensional questionnaire, recorded as a visual analogue scale (EQ-VAS), and the World Health Organization-5 Well-Being Index (WHO-5). At each visit, where possible the treating physician made and recorded their judgement of overall disease course (stable disease, slow progression, rapid progression) according to Behr et al. (2). Functional changes were categorized as stable/increased if FVC did not change or was improved by ≥0%, as a moderate decrease between > 0–10% and a significant decrease in case of > 10%.

All data were collected using an internet-based case report form (eCRF) with secure electronic data transfer to the central database. Quality measures included automated plausibility checks at data entry, statistical checks on data quality (focussing on missing values and outliers) as well as on-site monitoring and source data verification performed in the majority of centres (over 70%).

### Data analysis

Data were summarised by descriptive statistics including means and standard deviations and absolute and relative frequencies at baseline and each subsequent follow-up assessment. Baseline characteristics were compared by t-test for continuously distributed variables and Chi2 test for categorical variables between patients who were included in the analyses and excluded by missing QoL assessments. Linear mixed models were applied to investigate the change in QoL in follow-up with the study centres as cluster variable. Clinical characteristics such as lung function test at baseline (time invariant) and follow-up (time varying) were used to analyse possible associations with the change in QoL across time by linear mixed models. The association of mortality with QoL at the last available follow-up and change in QoL between baseline and last follow-up was analysed by Cox proportional hazard models. Hospitalisations for any reason and hospitalisations due to exacerbation in follow-up were investigated by linear mixed models. Multivariable regression models for mortality and hospitalisations included lung function test, the number of comorbidities, age and the physicians global judgement about disease activity as covariates. A *p*-value of lower than 0.05 was considered to be statistically significant.

Data were analysed with STATA 12.1 (StataCorp LP. Stata Statistical Software: Release 12. College Station, TX, USA).

## Results

### Patient characteristics

Of 879 patients, a total of 424 patients provided QoL data at baseline and at least at one follow-up visit, and were included in these analyses. Baseline characteristics of this cohort are presented in Table 1. Baseline characteristics for patients who were not included due to missing follow-up assessment of QoL -in comparison to the cohort reported here- are presented in Additional file 1: Table S4. For this the cohort, the patients mean age was 68.7 ± 9.1 years, 76.9% were male, and mean symptom duration was 3.7 ± 4.1 years, with 61.3% having disease for more than 6 months at registry enrolment; 60.4% of participants were former smokers with 38.0% never having smoked. Most patients (77.6%) had one or more comorbidities. At baseline, the IPF was judged by treating physicians as stable in 39.2% of patients, slowly progressing in 29.0% and rapidly progressing in 7.3%. Most patients were in NYHA functional class II/III at enrolment, with a mean 6MWD of 288 ± 200 m. Mean values for respiratory parameters were; FVC % predicted (75.9% ± 19.4), FEV_1_% (68.3% ± 17.4), and DL_CO_ % predicted (36.1% ± 15.9); 53.0% were in GAP index stage 2 and 28.2% in stage 3.

**Table 1:** Sociodemographic and clinical parameters of the patient cohort analysed

Patient characteristics	n (%) / mean (SD) *n* = 424
Female	98 (23.1%)
Age in years	68.7 (9.1)
Age at first symptom onset in years	64.9 (10.3)
Age at diagnosis in years	66.8 (9.7)
Duration since first symptoms in years	3.7 (4.1)
Disease duration in months	2.0 (2.7)
< 3 months	124 (29.5%)
3 to < 6 months	39 (9.3%)
More than 6 months	258 (61.3%)
Smoking status	
Never	161 (38.0%)
Former stopped	256 (60.4%)
Current	7 (1.7%)
Number of comorbidities	
None	95 (22.4%)
1	124 (29.3%)
2	107 (25.2%)
3	63 (14.9%)
4+	35 (8.3%)
NHYA	
I	28 (14.7%)
II	81 (42.6%)
III	76 (40.0%)
IV	5 (2.6%)
Six-minute walk distance (m)	287.7 (199.6)
% FEV_1_	68.3 (17.4)
% FVC	36.1 (15.9)
% DL_CO_	75.9 (19.4)
GAP index	4.7 (1.4)
Stage I	72 (18.8%)
Stage II	203 (53.0%)
Stage III	108 (28.2%)
Overall physician’s judgement of clinical course of IPF	
Stable disease	166 (39.2%)
Slow progression	123 (29.0%)
Rapid progression	31 (7.3%)
No judgement possible	104 (24.5%)
SGRQ	45.9 (19.7)
SGRQ symptoms	55.9 (21.0)
SGRQ activity	59.7 (23.6)
SGRQ impacts	34.8 (20.7)
UCSD-SOBQ	43.9 (28.8)
EQ-5D VAS	62.6 (18.5)
WHO-5	14.8 (5.7)

Values are n (%) or mean (SD)

*DL*_*CO*_ diffusing capacity of the lung for carbon monoxide, *EQ-5D VAS* EuroQol five-dimensional questionnaire, recorded as a visual analog scale, *FEV*_*1*_ Forced expiratory volume in 1 s, *FVC* forced vital capacity, *GAP index* Gender, Age, Physiology index, *IPF* idiopathic pulmonary fibrosis, *NYHA* New York Heart Association functional class, *SD* standard deviation, *SGRQ* St. George’s Respiratory Questionnaire, *USCD-SOBQ* University of California San Diego Shortness of Breath Questionnaire, *WHO-5* World Health Organization-5 Well-Being Index

At baseline the mean SGRQ total score was 45.9 ± 19.7, with higher scores for the SGRQ activity domain (59.7 ± 23.6) than symptom or impact domains. Patient reported breathlessness with a mean USCD-SOBQ score of 43.9 ± 28.8. Mean EQ-5D VAS scores were 62.6 ± 1 8.5 with mean WHO-5 scores of 14.8 ± 5.7.

### Change in QoL over time and associations to clinical parameters during follow up

**Fig. 1:**
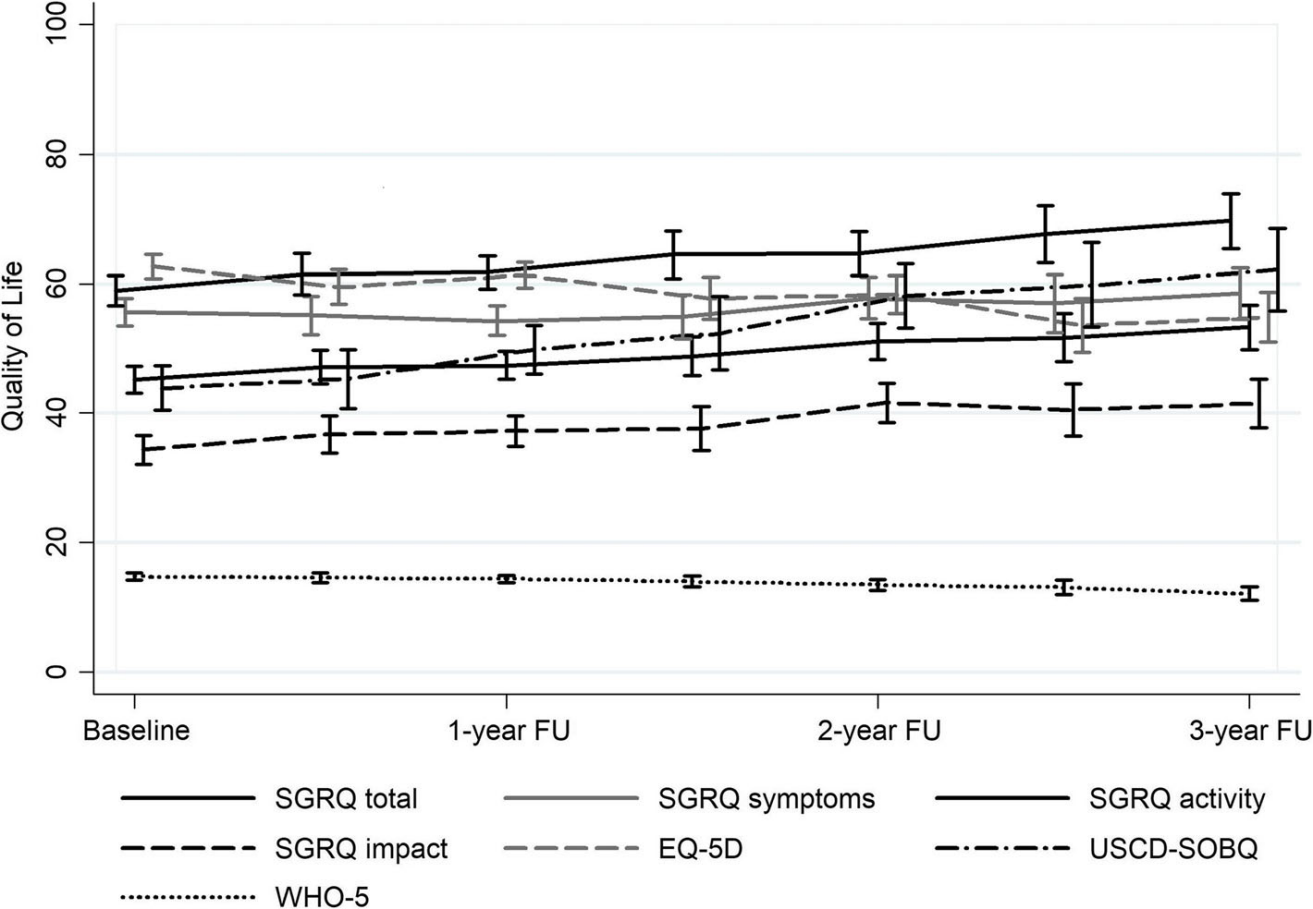
Change in QoL over 3 years of follow-up SGRQ (*p* < 0.001), SGRQ symptoms (*p* = 0.142), SGRQ activity (*p* < 0.001), SGRQ impacts (*p* < 0.001), EQ-5D VAS (*p* < 0.001), UCSD Shortness of breath (*p* < 0.001), WHO-5 (*p* < 0.001)) over 3 years of follow-up. EQ-5D VAS, EuroQol five-dimensional questionnaire, recorded as a visual analog scale; QoL, quality of life; SGRQ, St. George’s Respiratory Questionnaire; USCD-SOBQ, University of California San Diego Shortness of Breath Questionnaire; WHO-5, World Health Organization-5 Well-Being Index

The courses for QoL scores from baseline to last follow-up visit are shown in Fig. 1 (and in online data supplement; Additional file 2: Table S1). Longitudinal data showed a significant increase in SGRQ total scores (beta = 1.47, 95% CI: 1.17 to 1.76; *p* < 0.001) and UCSD-SOBQ score (beta = 3.63, 95% CI: 3.07 to 4.18; *p* < 0.001) per half-year between baseline and 36-months follow-up (indicating poorer QoL compared to baseline). EQ-5D VAS scores and WHO-5 scores also significantly decreased across time indicating a worsening QoL (Additional file 2: Table S1).

Details about the association between the different QoL measures and clinical characteristics at baseline and change in clinical parameters in follow-up are reported in Table 2. Female patients reported significant higher, i.e. poorer SGRQ scores during follow-up (beta = 5.42, 95% CI: 1.13 to 9.71; *p* = 0.013) compared to males. A higher NYHA stage, GAP index and a more severe assessment of the clinical course of the patient by the treating physician at baseline were associated with higher, i.e. worse SGRQ scores. The more impaired the lung function was at baseline, the higher were the SQRQ scores during follow-up. Patients with more than a 10% decrease of FVC % predicted and/or DL_CO_ % predicted within 1 year reported significantly worse SQRQ scores during follow-up compared to stable patients; FVC % predicted (beta = 9.08; 95% CI: 2.48 to 15.67; *p* = 0.007); DL_CO_ % predicted (beta = 7.79; 95% CI: 0.85 to 14.73; *p* = 0.028). Comparable results were observed for the other QoL measures (Table 2).

**Table 2:** Associations of QoL with clinical parameters at baseline and change in clinical parameters in follow-up

	SGRQ total			EQ 5D VAS			UCSD-SOBQ				WHO-5	
	beta	95% CI	*p*-value	beta	95% CI	*p*-value	beta	95% CI	*p*-value	beta	95% CI	*p*-value
Female sex	5.42	1.13; 9.71	0.013	−3.36	−6.90; 0.18	0.063	13.56	6.41; 20.72	< 0.001	− 1.47	−2.60; − 0.33	0.011
Age	0.00	−0.19; 0.20	0.963	−0.18	− 0.34; − 0.02	0.029	0.35	0.04; 0.66	0.026	−0.05	− 0.10; 0.00	0.074
Age at first symptom onset in years	−0.10	− 0.28; 0.08	0.285	− 0.08	− 0.22; 0.07	0.323	0.17	− 0.11; 0.46	0.238	− 0.03	− 0.07; 0.02	0.299
Age at diagnosis in years	−0.09	− 0.28; 0.09	0.320	− 0.08	− 0.23; 0.07	0.290	0.18	− 0.11; 0.48	0.227	− 0.02	− 0.07; 0.03	0.400
Duration since first symptoms in years	0.73	0.28; 1.18	0.002	−0.51	−0.89; − 0.13	0.008	0.71	− 0.04; 1.46	0.062	− 0.09	− 0.21; 0.03	0.139
Disease duration in months	1.36	0.70; 2.02	< 0.001	−1.10	− 1.65; −0.55	< 0.001	1.70	0.64; 2.77	0.002	−0.25	−0.43; − 0.08	0.005
< 3 months	1.00			1.00			1.00			1.00		
3 to < 6 months	−2.63	−9.30; 4.04	0.440	1.30	−4.28; 6.89	0.647	−4.48	−16.06; 7.11	0.449	0.54	−1.27; 2.34	0.560
More than 6 months	6.54	2.53; 10.55	0.001	−5.10	−8.43; − 1.76	0.003	8.43	1.74; 15.12	0.014	−0.68	−1.76; 0.40	0.215
Number of comorbidities												
None	1.00			1.00			1.00			1.00		
1	3.55	−1.37; 8.47	0.157	−5.19	−9.23; − 1.16	0.012	9.93	2.05; 17.80	0.013	−0.86	−2.16; 0.44	0.195
2	4.49	−0.63; 9.61	0.086	−6.46	−10.66; −2.26	0.003	10.11	1.88; 18.34	0.016	−1.02	−2.37; 0.33	0.140
3	5.87	−0.04; 11.78	0.051	−7.75	− 12.62; −2.88	0.002	15.37	5.74; 24.99	0.002	−1.98	−3.55; −0.41	0.013
4+	17.18	10.14; 24.23	< 0.001	−14.99	−20.88; −9.11	< 0.001	33.06	20.77; 45.35	< 0.001	−4.35	−6.24; −2.46	< 0.001
NHYA												
I	1.00			1.00			1.00			1.00		
II	16.07	9.16; 22.98	< 0.001	−12.61	−19.06; −6.15	< 0.001	22.28	10.78; 33.77	< 0.001	−1.86	−3.92; 0.21	0.078
III	26.95	19.97; 33.93	< 0.001	−18.00	−24.49; − 11.50	< 0.001	38.29	26.65; 49.92	< 0.001	−3.82	−5.90; − 1.75	< 0.001
IV	33.28	16.44; 50.13	< 0.001	−27.35	−41.90; − 12.81	< 0.001	51.63	24.63; 78.64	< 0.001	−8.69	−13.43; − 3.95	< 0.001
6MWD (m)	− 0.01	− 0.01; 0.00	0.217	0.00	0.00; 0.01	0.313	−0.01	− 0.03; 0.00	0.109	0.00	0.00; 0.00	0.423
FEV_1_% predicted	−0.55	−0.62; − 0.49	< 0.001	0.43	0.37; 0.49	< 0.001	− 0.80	− 0.91; − 0.70	< 0.001	0.09	0.07; 0.11	< 0.001
FVC % predicted	− 0.60	− 0.66; − 0.53	< 0.001	0.46	0.39; 0.53	< 0.001	−0.89	−1.00; − 0.78	< 0.001	0.11	0.09; 0.13	< 0.001
DL_CO_ % predicted	−0.26	− 0.31; − 0.20	< 0.001	0.26	0.20; 0.33	< 0.001	− 0.35	− 0.44; − 0.25	< 0.001	0.04	0.02; 0.06	< 0.001
Change in FVC % predicted between baseline and 1-year follow-up												
Stable/increase	1.00			1.00			1.00			1.00		
Decrease by 0 to 10%	3.71	−0.55; 7.97	0.087	−2.90	−6.58; 0.77	0.122	4.08	−3.13; 11.29	0.267	−0.51	−1.67; 0.65	0.393
Decrease by > 10%	9.08	2.48; 15.67	0.007	−8.07	−13.81; −2.33	0.006	11.24	0.32; 22.16	0.044	−1.30	−3.09; 0.50	0.156
Change in DL_CO_ % predicted between baseline and 1-year follow-up												
Stable/increase	1.00			1.00			1.00			1.00		
Decrease by 0 to 10%	5.46	0.85; 10.08	0.020	−0.41	−4.43; 3.60	0.841	10.46	2.85; 18.07	0.007	−0.37	−1.62; 0.88	0.561
Decrease by > 10%	7.79	0.85; 14.73	0.028	−3.93	−9.96; 2.10	0.201	10.74	−0.73; 22.21	0.067	−0.40	−2.28; 1.47	0.673
Change in FVC % predicted between baseline and last follow-up												
Stable/increase	1.00			1.00			1.00			1.00		
Decrease by 0 to 10%	3.44	−1.04; 7.92	0.133	−3.30	−7.08; 0.48	0.087	3.18	−4.45; 10.81	0.414	−0.22	−1.44; 0.99	0.719
Decrease by > 10%	7.47	2.11; 12.83	0.006	−6.50	−11.02; − 1.99	0.005	8.80	−0.15; 17.75	0.054	−0.87	−2.32; 0.59	0.242
Change in DL_CO_ % predicted between baseline and last follow-up												
Stable/increase	1.00			1.00			1.00			1.00		
Decrease by 0 to 10%	4.41	−0.52; 9.35	0.080	−1.42	−5.59; 2.75	0.505	11.09	2.94; 19.23	0.008	−0.44	−1.77; 0.89	0.518
Decrease by > 10%	5.10	−0.51; 10.72	0.075	−2.47	−7.18; 2.25	0.305	8.24	−0.88; 17.36	0.077	−0.30	−1.80; 1.21	0.701
GAP index	3.29	1.95; 4.64	< 0.001	−3.41	−4.50; −2.33	< 0.001	6.47	4.36; 8.58	< 0.001	−0.50	− 0.86; − 0.14	0.007
Stage I	1.00			1.00			1.00			1.00		
Stage II	5.69	0.74; 10.63	0.024	−7.04	−11.00; −3.08	< 0.001	16.51	8.69; 24.34	< 0.001	−0.92	−2.25; 0.41	0.177
Stage III	12.93	7.42; 18.45	< 0.001	−13.51	− 17.96; −9.07	< 0.001	25.78	17.08; 34.47	< 0.001	− 1.79	−3.27; −0.30	0.019
Overall physician’s judgement of clinical course of IPF												
Stable disease	1.00			1.00			1.00			1.00		
Slow progression	7.79	3.51; 12.06	< 0.001	−3.52	−7.14; 0.10	0.056	11.13	4.04; 18.21	0.002	−1.08	−2.23; 0.08	0.068
Rapid progression	13.16	6.22; 20.11	< 0.001	−9.52	−15.39; −3.65	0.001	19.89	8.32; 31.47	0.001	−2.72	−4.61; −0.83	0.005
No judgement possible	−0.39	−4.93; 4.15	0.865	1.33	−2.43; 5.09	0.487	1.72	−5.72; 9.15	0.651	−0.01	−1.23; 1.20	0.986

*6MWD* six-minute walk distance; beta, regression coefficient, *CI* confidence interval, *DL*_*CO*_ diffusing capacity of the lung for carbon monoxide, *EQ-5D VAS* EuroQol five-dimensional questionnaire, recorded as a visual analog scale, *FEV*_*1*_ Forced expiratory volume in 1 s, *FVC* forced vital capacity, *GAP index* Gender, Age, Physiology index, *IPF* idiopathic pulmonary fibrosis, *NYHA* New York Heart Association functional class, *QoL* quality of life, *SD* standard deviation, *SGRQ* St. George’s Respiratory Questionnaire, *USCD-SOBQ* University of California San Diego Shortness of Breath Questionnaire, *WHO-5* World Health Organization-5 Well-Being Index

**Fig. 2:**
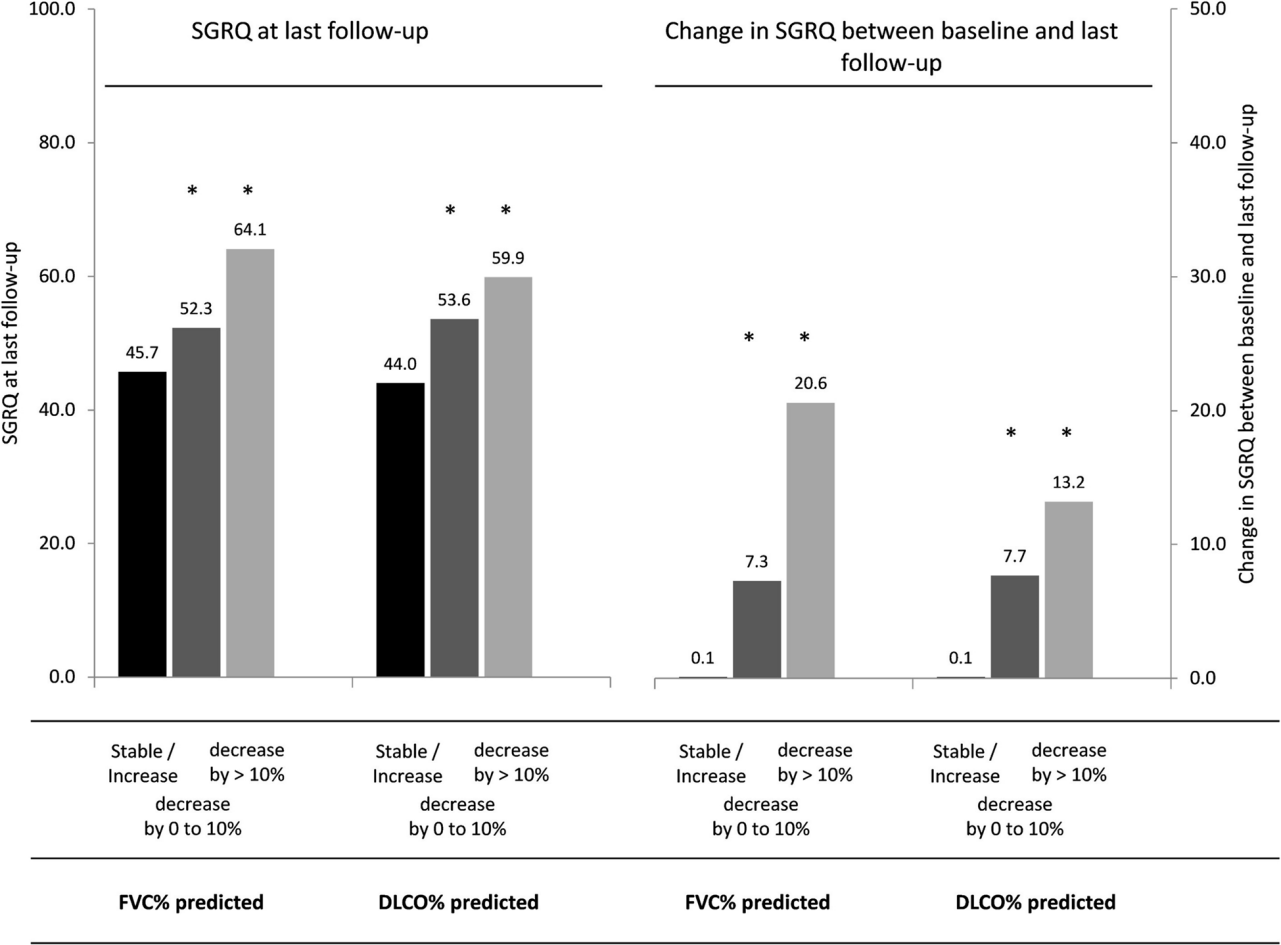
QoL at last follow-up visit and Change in QoL from baseline to last follow-up visit by change in lung function. * *p*-value < 0.05 in comparison to category ‘stable/ increase’ DL_CO_ % predicted: diffusing capacity of the lung for carbon monoxide % predicted; FVC % predicted, forced vital capacity % predicted; QoL, quality of life; SGRQ, St. George’s Respiratory Questionnaire

SGRQ at last follow-up, and change in SGRQ between baseline and last follow-up in association to lung functional changes within 1 year are shown in Fig. 2. More complete data for the SGRQ and those for all other QoL measures are presented in the online data supplement (Additional file 2: Table S2). The deterioration in SGRQ scores until last follow-up was associated with a worsening of FVC % predicted and DL_CO_ % predicted within 1 year after enrolment. In patients with stable or even improving, i.e. increasing FVC % predicted, SGRQ scores after 36 months follow-up remained relatively unchanged from baseline (mean − 0.1 units ‡ 21.1) with SGRQ at 36-months of 45.7 units ‡ SD 19.5. In contrast, compared to these patients, those patients with a decline of > 10% in FVC % predicted reported a significantly higher increase in SQRQ scores from baseline (mean 20.6 ‡19.1, beta = 20.71; 95% CI: 13.09 to 28.33; *p* < 0.001) and significantly higher SGRQ scores at last follow-up (mean = 64.1 ‡ 19.6, beta = 18.43; 95% CI: 10.98 to 25.87). There was also a non-significant trend towards a worse SGRQ for patients with a moderate lung functional decline compared to stable patients. A similar pattern was seen when comparing patients with a decline of > 10% in DL_CO_ % predicted with those with stable/improving DL_CO_ % predicted (Fig. 2). Similar associations were also seen for other QoL scores (SQRQ symptom, activity and impact domains, and UCSD-SOBQ, EQ-5D, WHO-5) (Online data supplement; Additional file 2: Table S2).

### Association of QoL and mortality

**Fig. 3:**
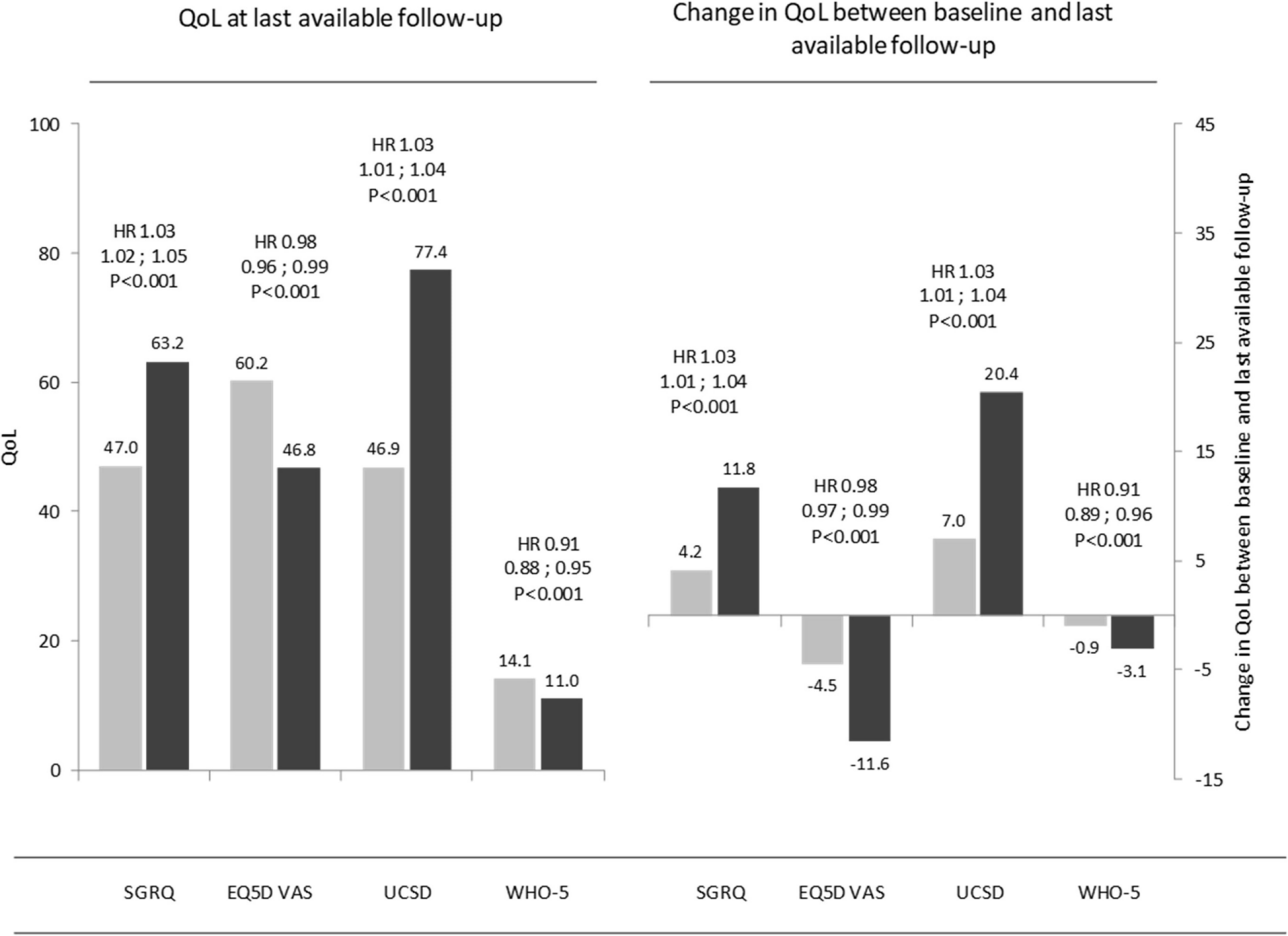
Association of QoL at last follow-up and change in QoL between baseline and last follow-up with mortality. Light grey and dark grey bares indicate patients who are censored and died during the observation period, respectively. Hazard ratios adjusted for QoL at baseline. EQ-5D VAS, EuroQol five-dimensional questionnaire, recorded as a visual analog scale; HR, Hazard ratio; QoL, quality of life; SGRQ, St. George’s Respiratory Questionnaire; USCD-SOBQ, University of California San Diego Shortness of Breath Questionnaire; WHO-5, World Health Organization-5 Well-Being Index

Over the study period there were 113 deaths (26.7% of all included patients). Univariate analysis found that change from baseline to last visit in any QoL outcome scores (i.e., SGRQ total score, UCSD-SOBQ, EQ-5D VAS, and WHO-5) were each predictive of mortality (Fig. 3). Patients who died during follow up in this cohort had a significant greater increase in SGRQ total scores from baseline to their last follow-up visit prior to death (mean 11.8 ± 18.6) compared to surviving patients (mean 4.2 ± 18.9; HR = 1.03; 95% CI: 1.02 to 1.05; *p* < 0.001). Changes from baseline in UCSD-SOBQ scores in patients who died and survivors were 7.0 ± 29.1 and 20.4 ± 1.5 respectively; HR = 1.03; 95% CI: 1.01 to 1.04; *p* < 0.001. In patients who died, the SGRQ and UCSD-SOBQ scores reported at last follow-up visit were all significantly higher (and EQ-5D VAS, and WHO-5 scores significantly lower) compared to surviving patients (Fig. 3).

After adjusting for change in FVC% predicted, age, number of comorbid diseases, hospitalisation during follow-up and the physician’s global assessment about the disease course, multivariate analysis found that a lower QoL at last available follow-up was significantly associated with mortality (Table 3). When evaluating mortality and SGRQ scores at last available follow-up, higher age (HR = 1.06; 95% CI: 1.03 to 1.09; *p* < 0.001) and a > 10% decline in FVC % predicted (HR = 2.34, 95% CI: 1.18 to 4.62; *p* = 0.015) were each associated with mortality. Furthermore, the higher the number of comorbid diseases the higher the risk for mortality (none versus one: HR = 2.40, 95% CI: 1.15 to 5.01; *p* = 0.02; none versus two: HR = 2.10, 95% CI: 1.02 to 4.32; *p* = 0.043; none versus three: HR = 2.78, 95% CI: 1.26 to 6.09; *p* = 0.011). Comparable results were found for the other QoL measures at last follow-up. In contrast, no significant associations between change from baseline to last available follow-up in QoL scores and mortality were observed in multivariate analysis (Table 3).

**Table 3:** Multivariable association of A) QoL at last available follow-up and B) change in QoL between Baseline and last available follow-up and clinical characteristics with mortality

	SGRQ total			EQ-5D VAS			UCSD-SOBQ			WHO-5		
	HR	95% CI	*p*-value	HR	95% CI	*p*-value	HR	95% CI	*p*-value	HR	95% CI	*p*-value
*A)* Multivariable model including QoL at last available follow-up												
QoL at last available follow-up ^a^	1.12	1.06; 1.18	< 0.001	0.91	0.87; 0.96	0.001	1.14	1.08; 1.20	< 0.001	0.83	0.69; 0.99	0.048
Age ^a^	1.32	1.14; 1.54	< 0.001	1.30	1.12; 1.51	0.001	1.27	1.09; 1.48	0.002	1.25	1.07; 1.46	0.006
Number of comorbidities												
0	1.00			1.00			1.00			1.00		
1	2.40	1.15; 5.01	0.02	1.93	0.98; 3.77	0.057	2.51	0.92; 6.81	0.071	2.03	0.93; 4.41	0.075
2	2.10	1.02; 4.32	0.043	1.62	0.83; 3.19	0.161	2.40	0.90; 6.40	0.079	2.24	1.05; 4.80	0.038
3	2.78	1.26; 6.09	0.011	2.15	1.02; 4.53	0.043	3.16	0.99; 10.05	0.052	2.31	0.97; 5.46	0.057
4+	1.95	0.69; 5.49	0.205	1.98	0.77; 5.07	0.155	1.30	0.29; 5.86	0.734	2.80	1.00; 7.86	0.051
Change in FVC % predicted												
Stable/increase	1.00			1.00			1.00			1.00		
Decrease by 0 to 10%	1.63	0.98; 2.71	0.058	1.60	0.97; 2.64	0.066	1.41	0.75; 2.64	0.286	1.75	1.01; 3.03	0.047
Decrease by > 10%	2.34	1.18; 4.62	0.015	2.29	1.16; 4.51	0.017	2.14	0.87; 5.26	0.096	2.70	1.32; 5.53	0.007
Hospitalisation	1.26	0.80; 1.97	0.322	1.54	0.98; 2.40	0.06	1.00	0.57; 1.74	0.994	1.29	0.81; 2.06	0.286
Overall physician’s judgement of clinical course of IPF												
Stable disease	1.00			1.00			1.00			1.00		
Slow progression	1.48	0.92; 2.38	0.106	1.70	1.08; 2.69	0.022	1.30	0.71; 2.37	0.399	1.91	1.16; 3.17	0.012
Rapid progression	0.97	0.46; 2.04	0.933	1.18	0.55; 2.53	0.674	1.06	0.46; 2.42	0.896	1.25	0.53; 2.95	0.617
No judgement possible	0.89	0.46; 1.74	0.743	0.98	0.52; 1.84	0.95	1.26	0.54; 2.93	0.588	0.94	0.47; 1.91	0.871
B) Multivariable model including change in QoL between baseline and last available follow-up												
Change in QoL between baseline and last available follow-up ^a^	1.05	0.98; 1.13	0.148	0.97	0.91; 1.04	0.364	1.07	0.98; 1.16	0.127	0.85	0.68; 1.07	0.165
Age ^a^	1.30	1.11; 1.52	0.001	1.31	1.12; 1.53	0.001	1.26	1.02; 1.56	0.031	1.26	1.07; 1.48	0.006
Number of comorbidities												
0	1.00			1.00			1.00			1.00		
1	1.98	0.97; 4.04	0.061	1.83	0.92; 3.63	0.084	2.03	0.71; 5.84	0.187	1.93	0.89; 4.20	0.096
2	1.85	0.90; 3.79	0.092	1.70	0.85; 3.40	0.135	2.01	0.65; 6.21	0.226	2.14	0.99; 4.63	0.054
3	2.40	1.10; 5.24	0.028	2.25	1.06; 4.76	0.035	3.68	1.17; 11.55	0.026	2.18	0.91; 5.23	0.082
4+	2.04	0.75; 5.57	0.165	2.27	0.87; 5.92	0.093	1.54	0.26; 9.06	0.634	2.99	1.09; 8.22	0.034
Change in FVC % predicted												
Stable/increase	1.00			1.00			1.00			1.00		
Decrease by 0 to 10%	1.69	1.02; 2.82	0.044	1.65	0.99; 2.76	0.055	1.11	0.57; 2.14	0.762	1.67	0.95; 2.91	0.073
Decrease by > 10%	2.16	1.08; 4.31	0.029	2.26	1.14; 4.50	0.02	1.72	0.71; 4.14	0.227	2.43	1.18; 5.00	0.015
Hospitalisation	1.35	0.85; 2.13	0.202	1.54	0.98; 2.42	0.059	1.30	0.68; 2.45	0.427	1.30	0.81; 2.08	0.285
Overall physician’s judgement of clinical course of IPF												
Stable disease	1.00			1.00			1.00			1.00		
Slow progression	1.69	1.05; 2.73	0.03	1.83	1.14; 2.94	0.012	1.38	0.67; 2.85	0.381	1.89	1.15; 3.12	0.013
Rapid progression	1.42	0.69; 2.91	0.341	1.63	0.76; 3.46	0.207	1.27	0.47; 3.41	0.636	1.38	0.61; 3.12	0.439
No judgement possible	0.97	0.49; 1.92	0.922	0.96	0.50; 1.83	0.902	1.24	0.52; 2.95	0.622	0.83	0.39; 1.77	0.635

*CI* confidence interval, *EQ-5D VAS* EuroQol five-dimensional questionnaire, recorded as a visual analog scale, *HR* hazard ratio, *FVC* forced vital capacity, *IPF* idiopathic pulmonary fibrosis, *QoL* quality of life, *SD* standard deviation, *SGRQ* St. George’s Respiratory Questionnaire, *USCD-SOBQ* University of California San Diego Shortness of Breath Questionnaire, *WHO-5* World Health Organization-5 Well-Being Index

^a^ HR for increase by 5 points on the QoL scale or by 5 years of age

**Table 4:** Multivariable association of QoL and clinical characteristics with any hospitalizations and hospitalizations due to exacerbations in follow-up

	SGRQ			EQ-5D VAS			UCSD-SOBQ			WHO-5		
	beta	95% CI	*p*-value	beta	95% CI	*p*-value	beta	95% CI	*p*-value	beta	95% CI	*p*-value
Any hospitalisation	3.95	0.35; 7.56	0.032	− 0.53	−3.70; 2.64	0.743	8.41	2.46; 14.37	0.006	−0.56	− 1.60; 0.48	0.292
Age	−0.04	− 0.26; 0.17	0.702	− 0.21	− 0.39; − 0.02	0.026	0.26	− 0.08; 0.61	0.129	− 0.05	− 0.11; 0.02	0.141
Number of comorbidities												
0	0.00			0.00			0.00			0.00		
1	−1.61	−7.37; 4.15	0.584	−0.40	−5.40; 4.60	0.875	0.31	−8.64; 9.26	0.946	0.29	−1.25; 1.83	0.714
2	1.82	−3.91; 7.56	0.533	−3.01	−8.19; 2.17	0.255	5.47	−3.72; 14.66	0.243	0.20	−1.36; 1.76	0.801
3	3.36	−2.91; 9.62	0.293	−4.07	−9.51; 1.38	0.143	7.11	−2.70; 16.93	0.155	−0.96	−2.63; 0.71	0.259
4+	11.42	4.99; 17.85	0.001	−10.18	− 16.28; −4.07	0.001	22.96	11.27; 34.65	< 0.001	−2.64	−5.02; −0.27	0.029
Change in FVC % predicted												
Stable/increase	0.00			0.00			0.00			0.00		
Decrease by 0 to 10%	3.70	−0.10; 7.51	0.057	−2.93	−6.17; 0.32	0.077	4.60	−1.67; 10.87	0.151	−0.51	−1.62; 0.61	0.375
Decrease by > 10%	8.08	2.23; 13.93	0.007	−6.11	−11.14; − 1.08	0.017	8.91	−0.23; 18.05	0.056	−0.97	−2.59; 0.66	0.242
Overall physician’s judgement of clinical course of IPF												
Stable disease	0.00			0.00			0.00			0.00		
Slow progression	7.15	4.88; 9.42	< 0.001	−7.24	− 10.05; −4.44	< 0.001	10.65	5.96; 15.35	< 0.001	−1.62	−2.34; − 0.89	< 0.001
Rapid progression	22.68	16.83; 28.52	< 0.001	− 26.37	−32.73; − 20.01	< 0.001	33.57	24.66; 42.48	< 0.001	−6.35	−8.31; −4.40	< 0.001
No judgement possible	3.02	0.33; 5.70	0.027	−2.15	−5.16; 0.87	0.163	1.70	−2.96; 6.35	0.475	−1.25	−2.11; −0.40	0.004
Hospitalisation by exacerbation	5.35	0.64; 10.07	0.026	−1.56	−5.78; 2.65	0.468	10.89	2.72; 19.05	0.009	−0.78	−2.32; 0.76	0.320
Age	0.04	−0.24; 0.31	0.791	−0.37	− 0.59; − 0.15	0.001	0.40	− 0.02; 0.82	0.062	− 0.04	−0.12; 0.04	0.383
Number of comorbidities												
0	0.00			0.00			0.00			0.00		
1	−0.33	−6.99; 6.32	0.922	−1.35	−7.22; 4.51	0.651	1.41	−8.79; 11.61	0.786	0.23	−1.58; 2.04	0.804
2	2.93	−3.86; 9.72	0.397	−2.43	−8.49; 3.64	0.433	6.77	−4.36; 17.89	0.233	−0.09	−2.05; 1.88	0.930
3	3.52	−3.89; 10.93	0.352	−1.79	−8.17; 4.60	0.583	7.09	−5.08; 19.27	0.253	−0.94	−3.15; 1.26	0.403
4+	13.15	5.95; 20.36	< 0.001	−8.90	−15.99; − 1.82	0.014	21.07	8.29; 33.84	0.001	−2.60	−5.56; 0.36	0.085
Change in FVC % predicted												
Stable/increase	0.00			0.00			0.00			0.00		
Decrease by 0 to 10%	4.08	−0.73; 8.88	0.096	−4.12	−8.39; 0.15	0.059	6.63	−1.24; 14.50	0.099	−0.75	−2.17; 0.67	0.300
Decrease by > 10%	7.49	0.66; 14.32	0.032	−4.05	−10.00; 1.90	0.182	9.98	−0.91; 20.86	0.072	−1.04	−2.79; 0.72	0.247
Overall physician’s judgement of clinical course of IPF												
Stable disease	0.00			0.00			0.00			0.00		
Slow progression	6.65	3.84; 9.46	< 0.001	−8.90	−12.49; −5.31	< 0.001	9.98	3.38; 16.58	0.003	−1.63	−2.55; −0.70	0.001
Rapid progression	24.23	17.19; 31.26	< 0.001	−27.75	−36.15; −19.34	< 0.001	33.78	22.82; 44.75	< 0.001	−6.67	−9.06; −4.29	< 0.001
No judgement possible	2.36	−0.77; 5.49	0.139	−1.16	−4.97; 2.66	0.552	−0.76	−6.46; 4.94	0.794	−0.98	−2.00; 0.03	0.058

*beta* regression coefficient, *CI* confidence interval, *EQ-5D VAS* EuroQol five-dimensional questionnaire, recorded as a visual analog scale, *FVC* forced vital capacity, *IPF* idiopathic pulmonary fibrosis, *QoL* quality of life, *SD* standard deviation, *SGRQ* St. George’s Respiratory Questionnaire, *USCD-SOBQ* University of California San Diego Shortness of Breath Questionnaire, *WHO-5* World Health Organization-5 Well-Being Index

### Association of QoL and hospitalisation and exacerbations

**Fig. 4:**
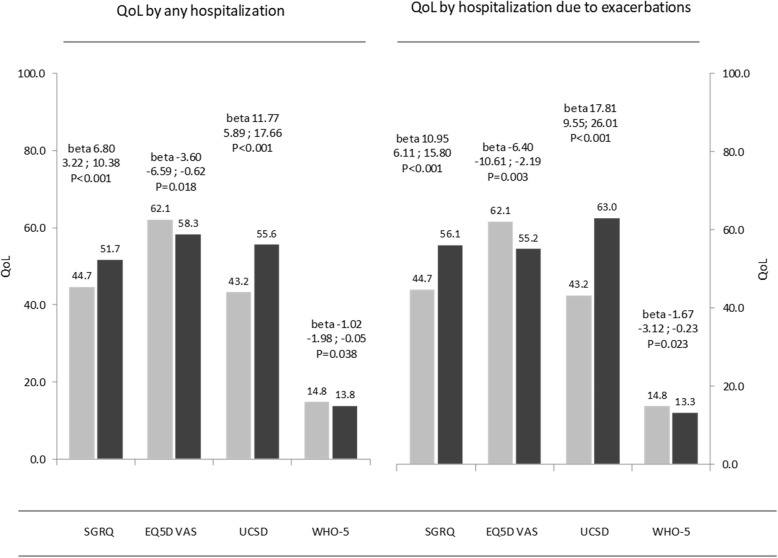
Mean QoL scores for patients who were hospitalized (dark grey bares) during follow-up compared to patients who were not hospitalized (light grey bares). EQ-5D VAS, EuroQol five-dimensional questionnaire, recorded as a visual analog scale; QoL, quality of life; SGRQ, St. George’s Respiratory Questionnaire; USCD-SOBQ, University of California San Diego Shortness of Breath Questionnaire; WHO-5, World Health Organization-5 Well-Being Index

Over the study period 227 patients were hospitalised for any cause and 31 hospitalised for exacerbations. Univariate and multivariate analysis found that the mean SGRQ and UCSD-SOBQ scores across the follow-up period were all significantly higher (and for univariate analyses EQ-5D VAS, and WHO-5 score significantly lower) in hospitalised patients compared to non-hospitalised patients, with the highest mean SGRQ and UCSD-SOBQ scores throughout follow-up (and lowest EQ-5D VAS and WHO-5 scores) reported in those hospitalised for IPF (Table 4, Fig. 4). Furthermore, across the follow-up period an overall trend was observed, in which successively greater increases in mean SGRQ and UCSD-SOBQ scores and successively greater reductions in EQ-5D VAS and WHO-5 scores were reported by patients hospitalised on 1, 2, 3 and ≥ 4 separate occasions (Online data supplement; Additional file 3: Figure S1).

Hospitalisation had an adverse impact upon subsequent QoL. Following hospitalisation, significantly higher mean SGRQ scores (total scores and activity and impacts domain scores) and higher mean UCSD-SOBQ scores were reported at the subsequent follow-up visit compared to those reported at the visit prior to hospitalisation (and significantly lower mean EQ-5D VAS and WHO-5 scores) (Online data supplement; Additional file 2: Table S3).

## Discussion

According to the present data, collected from a representative setting of centres with expertise in the management of patients with IPF, a close relationship between QoL and clinical outcomes were found; including clinically meaningful changes in lung function, comorbidities, disease duration and clinical course of IPF (including hospitalisation, acute exacerbations and mortality). For these analyses, we used a range of QoL assessments which, although not originally designed for IPF, have data to support their validity in this patient population, as shown in our previous report and by others [6, 7, 9, 15–18]. The optimal tool for QoL assessment in IPF patients remains to be determined, with an adaptation of the SGRQ specifically for use in IPF not yet fully validated or in widespread use [19], while other more focused IPF QoL-measures are also in use or being developed for use (e.g. the Kings’ Brief Interstitial Lung Disease health status [K-BILD] and the ‘A Tool to Assess Quality of Life in Idiopathic Pulmonary Fibrosis [ATAQ-IPFcA] questionnaires) [9, 20, 21]. It is likely that, while subtle differences may exist, most current tools assess QoL with similar sensitivity and with high or very high correlation between most questionnaires [7, 9].

To our knowledge, the only previous report on longitudinal assessments of QoL in IPF patients in clinical practice comes from the Australian IPF registry in a somewhat smaller dataset [6]. In that study, a strong relationship between decline in FVC % predicted and decline in QoL was shown, with QoL impairment mainly due to dyspnoea (measured using the SGRQ and UCSD-SOBQ scores) although cough and depression (assessed by a VAS scale and the Hospital Anxiety and Depression Scale (HADS)) also contributed [6]. While in that study, no association between baseline QoL measures and mortality was found, a more recent report from the Australian registry indicates that in those patients with mild physiological impairment (FVC ≥ 80%), baseline SGRQ and UCSD-SOBQ scores are predictive of mortality, with HRs of 1.11 (95% CI: 1.06 to 1.15) and 1.23 (95% CI: 1.13 to 1.33) respectively (*p* < 0.001 for both) [22]. In contrast to the Australian Registry, we found a significant relationship between lower QoL at last available follow-up but no significant association (on multivariable analysis) between mortality and change in QoL for the total IPF population. Number of comorbidities as well as hospitalisations were driving this QoL decline. Notably, significant changes in both FVC and DLco % predicted values closely reflected changes in QoL indicating that stabilising lung function may also halt decline of QoL in IPF.

The overall pattern we observed across the cohort was a longitudinal decline in QoL. How then can we seek to improve upon or at least maintain patient QoL in clinical practice? At present, only sparse data on the long-term effects of available therapies on QoL outcomes exist. While disease stabilisation by antifibrotic therapies may offer some potential in maintaining QoL (or retarding decline), available data to support this goal remain limited. In the pivotal phase 3 trials evaluating nintedanib and pirfenidone (each versus placebo), no significant differences in changes in SGRQ scores (from baseline to 52 weeks) were seen between the treatment and placebo arms [23, 24] a finding which could be due to side effects or to short observation times. Furthermore, this has also to be interpreted in light of the here reported differences in associations of QoL to lung functional decline which was statistically significant comparing stable to significantly progressing patients, i.e. with a decrease of FVC > 10%. However, the difference to moderately progressive patients, i.e. a decline between 0 and 10% was only by trend. In the INPULSIS trials for example adjusted absolute mean change from baseline in FVC% was also within this range with − 2.8 for nintedanib and − 6.0 for placebo. Still, a subsequent pooled analysis of the TOMORROW and INPULSIS trials comparing nintedanib vs. placebo has shown that the reduction in annual FVC % predicted decline seen in the nintedanib arms was accompanied by lower increases in SGRQ compared to placebo (with a mean difference in adjusted mean change from baseline of − 2.05 [95% CI: –3.59 to − 0.50]; *p* < 0.0095) [25]. In the step-IPF study, sildenafil was reported to positively impact upon QoL in IPF patients with severe impairment of gas exchange (i.e. DLco ≤35%) [26]. In a recent randomised controlled trial using nintedanib and sildenafil in combination (INSTAGE study) a positive, while non-significant trend on QoL was observed, but conclusive data is still lacking [27]. On balance, it is likely that optimising pharmacotherapy in IPF patients (and reducing decline in lung function) may also assist in improving QoL in IPF patients. As groups in cohort studies like ours, e.g. with and without antifibrotic therapies, inevitably differ in their clinical characteristics comparisons of therapy effects on QoL in registries may be subject to bias by indication. We therefore refrained from performing comparative analyses with regards to therapy, which however should be assessed in future research.

Non-pharmacotherapy treatments are also important. As physical activity is a strong predictor of IPF disease course, methods to increase physical activity e.g., pulmonary rehabilitation are important in prolonging a more positive life-span, in particular as data suggests that pulmonary rehabilitation may increase QoL [28–30]. Allied to this, greater attention to assessment and treatment of comorbid conditions may improve or at least alleviate longitudinal decline in QoL, although as of yet this remains unproven [4]. For several patients, early palliative care may be an appropriate approach [5, 12].

Our study has a number of limitations (above that of using non-IPF specific QoL measures as discussed above). Typically for data collection under clinical practice conditions, sites showed considerable variance in terms of number of documented visits and visit time-points, and in terms of completeness of data (each depending on their routine clinical schedule) including QoL reports. Furthermore, a potential bias cannot be excluded, such as underreporting or underdiagnosing of subacute deterioration and any influence of social factors (e.g., family situation) and other aspects external to the disease process. A high number of patients enrolled were excluded from the present analyses due to incomplete data of longitudinal QoL assessment. As some differences had been noted between the group of in- and excluded patients, e.g. a more severe disease and a higher mean number of comorbidities, a bias of our results toward patients with a more severe disease course cannot be excluded. In addition, not every patient completed each of the QoL questionnaires at least yearly as stipulated. However, the relatively large numbers of included patients and completed QoL measures in connection with a long follow-up period do provide a substantially large sample and exposure time from which we found significant differences in a number of outcomes, and association of QoL changes with clinical status. A final limitation is that we have not included potential effects of treatment (including use of anti-fibrotic agents) in the present analyses and cannot report on benefits of such treatment.

## Conclusions

In conclusion, we found strong associations of changes of QoL to important outcomes in IPF; principally decline in lung function, hospitalisation for acute exacerbations and mortality. Pharmacological and non-pharmacological therapies used to reduce these clinically important events should also aim to maintain or even improve QoL as assessed by these equally important patient-reported outcomes.

## Additional files

Additional file 1: **Table S4.** Sociodemographic and clinical parameters of the study sample comparing patients who were not included due to missing follow-up assessment of QoL in comparison to the cohort included into the analyses. (DOCX 24 kb)

Additional file 2: **Table S1.** Quality of life scores at each follow-up visit and change in quality of life over the 3-year follow-up. **Table S2.** Quality of life scores at last follow-up and change in quality of life between baseline and last follow-up by change in lung function parameters within 1 year after baseline. **Table S3.** Quality of life scores at visit prior to hospitalisation and subsequent visit after hospitalisation in follow-up. (DOCX 40 kb)

Additional file 3: **Figure S1.** Mean difference in QoL scores for patients with one, two, three and more than three hospitalizations compared to patients who were not hospitalized during the follow-up. (EQ-5D VAS, EuroQol five-dimensional questionnaire, recorded as a visual analog scale; QoL, quality of life; SGRQ, St. George’s Respiratory Questionnaire; USCD-SOBQ, University of California San Diego Shortness of Breath Questionnaire; WHO-5, World Health Organization-5 Well-Being Index). (DOCX 110 kb)

## Abbreviations

6MWD: Six-minute walk distance
DL_CO_: Diffusing capacity of the lung for carbon monoxide
EQ-5D VAS: EuroQol five-dimensional questionnaire, recorded as a visual analog scale
FEV_1_: Forced expiratory volume in 1 s
FVC % predicted: Forced vital capacity % predicted
GAP index: Gender, Age, Physiology index
NYHA: New York Heart Association functional class
SGRQ: St. George’s Respiratory Questionnaire
USCD-SOBQ: University of California San Diego Shortness of Breath Questionnaire
WHO-5: World Health Organization-5 Well-Being Index
