# Supplementary material for: The clinical course of idiopathic pulmonary fibrosis and its association to quality of life over time: longitudinal data from the INSIGHTS-IPF registry
Source: Respir Res. 2019 Mar 15;20:59. doi: 10.1186/s12931-019-1020-3 (PMC6420774; doi:10.1186/s12931-019-1020-3)
Supplement: Supplementary file 1 — Table S4. Sociodemographic and clinical parameters of the study sample comparing patients who were not included due to missing follow-up assessment of QoL in comparison to the cohort included into the analyses. (DOCX 24 kb) [file 12931_2019_1020_MOESM1_ESM.docx]

Table S4: Sociodemographic and clinical parameters of the study sample comparing patients who were not included due to missing follow-up assessment of QoL in comparison to the cohort included into the analyses

|  |  |  |  |  |
| --- | --- | --- | --- | --- |
|  |  |  |  |  |
|  |  | **Patients not included into analyses** | **Patients included into analyses** |  |
|  |  |  |  |  |
|  |  | n (%) / mean(sd) | n (%) / mean(sd) | p value |
|  |  | n=455 | n=424 |  |
|  |  |  |  |  |
|  |  |  |  |  |
| Female | | 77 (16.9%) | 98 (23.1%) | 0.022 |
| Age in years | | 70.5 (8.8) | 68.7 (9.1) | 0.003 |
|  |  |  |  |  |
| Age at first symptom onset in years | | 66.7 (10.3) | 64.9 (10.3) | 0.015 |
| Age at diagnosis in years | | 68.8 (9.7) | 66.8 (9.7) | 0.003 |
|  |  |  |  |  |
| Duration since first symptoms in years | | 3.5 (4.1) | 3.7 (4.1) | 0.714 |
| Disease duration in month | | 1.8 (3.5) | 2.0 (2.7) | 0.337 |
|  | < 3 months | 166 (36.6%) | 124 (29.5%) | 0.035 |
|  | 3 to <6 months | 49 (10.8%) | 39 (9.3%) |  |
|  | more than 6 months | 239 (52.6%) | 258 (61.3%) |  |
|  |  |  |  |  |
| Smoking status | |  |  | 0.240 |
|  | never | 148 (32.5%) | 161 (38.0%) |  |
|  | former stopped | 299 (65.7%) | 256 (60.4%) |  |
|  | current | 8 (1.8%) | 7 (1.7%) |  |
|  |  |  |  |  |
| Number of comorbidities | |  |  | 0.001 |
|  | none | 87 (19.1%) | 95 (22.4%) |  |
|  | 1 | 107 (23.5%) | 124 (29.3%) |  |
|  | 2 | 107 (23.5%) | 107 (25.2%) |  |
|  | 3 | 76 (16.7%) | 63 (14.9%) |  |
|  | 4+ | 78 (17.1%) | 35 (8.3%) |  |
|  |  |  |  |  |
| NHYA | |  |  | 0.012 |
|  | I | 17 (9.3%) | 28 (14.7%) |  |
|  | II | 60 (32.8%) | 81 (42.6%) |  |
|  | III | 93 (50.8%) | 76 (40.0%) |  |
|  | IV | 13 (7.1%) | 5 (2.6%) |  |
|  |  |  |  |  |
| Six-minute walk distance in meter | | 265.7 (181.5) | 287.7 (199.6) | 0.093 |
|  |  |  |  |  |
| % FEV_1_ | | 67.3 (18.8) | 68.3 (17.4) | 0.430 |
| % FVC | | 35.4 (16.9) | 36.1 (15.9) | 0.579 |
| % DL_CO_ | | 75.1 (19.4) | 75.9 (19.4) | 0.558 |
|  |  |  |  |  |
| GAP index | | 4.9 (1.5) | 4.7 (1.4) | 0.093 |
|  | Stage I | 82 (19.2%) | 72 (18.8%) | 0.053 |
|  | Stage II | 192 (44.9%) | 203 (53.0%) |  |
|  | Stage III | 154 (36.0%) | 108 (28.2%) |  |
|  |  |  |  |  |
| Overall physician’s judgement of clinical course of IPF | |  |  | 0.127 |
|  | stable disease | 179 (39.3%) | 166 (39.2%) |  |
|  | slow progression | 107 (23.5%) | 123 (29.0%) |  |
|  | rapid progression | 49 (10.8%) | 31 (7.3%) |  |
|  | no judgement possible | 120 (26.4%) | 104 (24.5%) |  |
|  |  |  |  |  |
| EQ5D VAS | | 56.6 (20.7) | 62.6 (18.5) | <0.001 |
|  |  |  |  |  |
| WHO-5 | | 12.7 (6.3) | 14.8 (5.7) | <0.001 |
|  |  |  |  |  |
| SGRQ | | 50.9 (21.6) | 45.9 (19.7) | 0.001 |
| SGRQ symptoms | | 58.6 (21.4) | 55.9 (21.0) | 0.094 |
| SGRQ activity | | 64.7 (25.0) | 59.7 (23.6) | 0.006 |
| SGRQ impacts | | 40.7 (22.8) | 34.8 (20.7) | 0.001 |
|  |  |  |  |  |
| UCSD Shortness of breath | | 51.9 (34.2) | 43.9 (28.8) | 0.006 |
|  |  |  |  |  |
| sd = standard deviation; VAS = visual analogue scale | | | | |
