# Supplementary material for: The clinical course of idiopathic pulmonary fibrosis and its association to quality of life over time: longitudinal data from the INSIGHTS-IPF registry
Source: Respir Res. 2019 Mar 15;20:59. doi: 10.1186/s12931-019-1020-3 (PMC6420774; doi:10.1186/s12931-019-1020-3)
Supplement: Supplementary file 2 — Table S1. Quality of life scores at each follow-up visit and change in quality of life over the 3-year follow-up. Table S2. Quality of life scores at last follow-up and change in quality of life between baseline and last follow-up by change in lung function parameters within 1 year after baseline. Table S3. Quality of life scores at visit prior to hospitalisation and subsequent visit after hospitalisation in follow-up. (DOCX 40 kb) [file 12931_2019_1020_MOESM2_ESM.docx]

**Additional File 2**

# Table S1: Quality of life scores at each follow-up visit and change in quality of life over the 3-year follow-up

|  | **Baseline** | | | **1/2 year follow-up** | | | | **1 year follow-up** | | | **1 1/2 year follow-up** | | | **2 year follow-up** | | | **2 1/2 year follow-up** | | | | **3 year follow-up** | | |  | |  |  | |  |
| --- | --- | --- | --- | --- | --- | --- | --- | --- | --- | --- | --- | --- | --- | --- | --- | --- | --- | --- | --- | --- | --- | --- | --- | --- | --- | --- | --- | --- | --- |
|  | **N** | **mean** | **SD** |  | **N** | **mean** | **SD** | **N** | **mean** | **SD** | **N** | **mean** | **SD** | **N** | **mean** | **SD** | | **N** | **mean** | **SD** | **N** | **mean** | **SD** | | **beta†** | **95% CI** | | **p-value** | |
| **SGRQ** | 401 | 45.9 | 19.7 |  | 191 | 52.3 | 19.2 | 502 | 47.2 | 21.5 | 163 | 47.3 | 18.9 | 275 | 49.6 | 24.5 | | 124 | 48.8 | 21.4 | 196 | 46.3 | 22.4 | | 1.47 | 1.17 to 1.76 | | <0.001 | |
| **SGRQ symptoms** | 395 | 55.9 | 21.0 |  | 190 | 58.1 | 18.9 | 511 | 54.6 | 23.5 | 162 | 54.9 | 20.8 | 270 | 59.6 | 22.8 | | 107 | 56.8 | 23.2 | 211 | 53.7 | 23.2 | | 0.54 | 0.19 to 0.90 | | 0.003 | |
| **SGRQ activity** | 404 | 59.7 | 23.6 |  | 187 | 67.4 | 21.8 | 503 | 61.1 | 26.1 | 164 | 63.2 | 21.6 | 273 | 62.3 | 28.2 | | 128 | 63.2 | 24.6 | 201 | 57.9 | 28.4 | | 1.78 | 1.42 to 2.14 | | <0.001 | |
| **SGRQ impacts** | 355 | 34.8 | 20.7 |  | 179 | 41.4 | 20.8 | 484 | 37.5 | 22.3 | 153 | 35.1 | 20.2 | 259 | 40.9 | 26.2 | | 126 | 37.4 | 22.7 | 195 | 34.7 | 22.4 | | 1.41 | 1.07 to 1.75 | | <0.001 | |
| **EQ-5D VAS** | 420 | 62.6 | 18.5 |  | 210 | 57.7 | 20.6 | 550 | 61.9 | 20.5 | 180 | 59.3 | 18.5 | 291 | 60.1 | 21.1 | | 126 | 54.3 | 18.7 | 223 | 60.3 | 21.1 | | -1.51 | -1.86 to -1.16 | | <0.001 | |
| **UCSD-SOBQ** | 271 | 43.9 | 28.8 |  | 128 | 50.9 | 30.0 | 323 | 50.9 | 33.5 | 105 | 49.8 | 25.6 | 176 | 54.6 | 34.7 | | 72 | 57.1 | 28.2 | 126 | 48.0 | 34.7 | | 3.63 | 3.07 to 4.18 | | <0.001 | |
| **WHO-5** | 407 | 14.8 | 5.7 |  | 193 | 14.2 | 5.7 | 510 | 14.3 | 5.7 | 180 | 14.4 | 5.4 | 274 | 14.0 | 6.7 | | 123 | 13.7 | 6.1 | 201 | 13.2 | 6.5 | | -0.43 | -0.52 to -0.33 | | <0.001 | |

†Change in quality of life per half-year follow-up estimated by a linear mixed model

beta, regression coefficient; CI, confidence interval; EQ-5D VAS, EuroQol five-dimensional questionnaire, recorded as a visual analog scale; SD, standard deviation; SGRQ, St. George’s Respiratory Questionnaire; USCD-SOBQ, University of California San Diego Shortness of Breath Questionnaire; WHO-5, World Health Organization-5 Well-Being Index.

# Table S2: Quality of life scores at last follow-up and change in quality of life between baseline and last follow-up by change in lung function parameters within one year after baseline

|  | **stable/increase** | |  | **decrease by 0 to 10%** | | | | |  | **decrease by >10%** | | | | |
| --- | --- | --- | --- | --- | --- | --- | --- | --- | --- | --- | --- | --- | --- | --- |
|  | **mean** | **SD** |  | **mean** | **SD** | **beta** | **95% CI** | **p-value** |  | **mean** | **SD** | **beta** | **95% CI** | **p-value** |
| ***QoL at last follow-up by change FVC% predicted between baseline and 1-year follow-up*** | | | | | | | | | | | | | | |
| **SGRQ** | 45.7 | 19.5 |  | 52.3 | 21.3 | 6.61 | 1.60; 11.61 | 0.010 |  | 64.1 | 19.6 | 18.43 | 10.98; 25.87 | <0.001 |
| **SGRQ symptoms** | 50.2 | 24.1 |  | 58.0 | 22.4 | 7.79 | 2.00; 13.58 | 0.009 |  | 71.8 | 16.9 | 21.55 | 14.35; 28.75 | <0.001 |
| **SGRQ activity** | 63.9 | 22.4 |  | 66.1 | 24.1 | 2.22 | -3.49; 7.92 | 0.445 |  | 74.0 | 20.3 | 10.17 | 2.23; 18.10 | 0.012 |
| **SGRQ impacts** | 33.8 | 20.6 |  | 42.4 | 23.8 | 8.60 | 3.10; 14.10 | 0.002 |  | 56.1 | 23.1 | 22.31 | 13.68; 30.94 | <0.001 |
| **EQ-5D VAS** | 62.8 | 18.2 |  | 55.5 | 22.5 | -7.24 | -11.93; -2.55 | 0.003 |  | 45.9 | 20.6 | -16.89 | -24.09; -9.69 | <0.001 |
| **UCSD-SOBQ** | 44.7 | 30.6 |  | 57.0 | 33.0 | 12.23 | 2.15; 22.32 | 0.018 |  | 74.7 | 28.2 | 29.99 | 16.50; 43.48 | <0.001 |
| **WHO-5** | 14.9 | 5.3 |  | 13.0 | 6.5 | -1.90 | -3.31; -0.48 | 0.009 |  | 11.2 | 5.8 | -3.67 | -5.80; -1.54 | 0.001 |
|  |  |  |  |  |  |  |  |  |  |  |  |  |  |  |
| ***Change in QoL between baseline and last follow-up by change FVC% predicted between baseline and 1-year follow-up*** | | | | | | | | | | | | | | |
| **SGRQ** | -0.1 | 21.1 |  | 7.2 | 17.0 | 7.27 | 2.25; 12.28 | 0.005 |  | 20.6 | 19.1 | 20.71 | 13.09; 28.33 | <0.001 |
| **SGRQ symptoms** | -2.6 | 26.8 |  | -0.5 | 20.8 | 2.09 | -4.14; 8.31 | 0.510 |  | 14.2 | 20.6 | 16.85 | 8.21; 25.50 | <0.001 |
| **SGRQ activity** | 3.0 | 24.2 |  | 8.3 | 20.8 | 5.33 | -0.50; 11.16 | 0.073 |  | 20.0 | 23.5 | 17.08 | 8.02; 26.15 | <0.001 |
| **SGRQ impacts** | -0.1 | 22.8 |  | 7.9 | 18.7 | 8.05 | 2.18; 13.92 | 0.007 |  | 20.9 | 20.4 | 21.05 | 12.24; 29.85 | <0.001 |
| **EQ-5D VAS** | 1.7 | 26.8 |  | -7.6 | 22.8 | -9.24 | -15.24; -3.24 | 0.003 |  | -20.8 | 20.5 | -22.53 | -30.73; -14.33 | <0.001 |
| **UCSD-SOBQ** | -2.3 | 36.2 |  | 12.6 | 24.4 | 14.87 | 2.72; 27.01 | 0.017 |  | 31.9 | 29.2 | 34.11 | 17.60; 50.62 | <0.001 |
| **WHO-5** | 0.9 | 6.5 |  | -1.9 | 5.5 | -2.80 | -4.34; -1.25 | <0.001 |  | -4.4 | 5.3 | -5.30 | -7.45; -3.16 | <0.001 |
|  |  |  |  |  |  |  |  |  |  |  |  |  |  |  |
| ***QoL at last follow-up by change DLCO% predicted between baseline and 1-year follow-up*** | | | | | | | | | | | | | | |
| **SGRQ** | 44.0 | 19.7 |  | 53.6 | 20.7 | 9.60 | 4.20; 15.00 | 0.001 |  | 59.9 | 21.2 | 15.90 | 7.62; 24.18 | <0.001 |
| **SGRQ symptoms** | 52.2 | 25.9 |  | 58.1 | 22.0 | 5.91 | -0.72; 12.53 | 0.080 |  | 64.4 | 20.9 | 12.17 | 3.24; 21.09 | 0.008 |
| **SGRQ activity** | 59.0 | 22.7 |  | 68.3 | 22.5 | 9.29 | 3.21; 15.36 | 0.003 |  | 72.6 | 21.3 | 13.65 | 4.98; 22.32 | 0.002 |
| **SGRQ impacts** | 33.8 | 21.3 |  | 43.7 | 23.8 | 9.90 | 3.93; 15.87 | 0.001 |  | 52.0 | 23.0 | 18.22 | 8.64; 27.79 | <0.001 |
| **EQ-5D VAS** | 60.8 | 19.2 |  | 55.7 | 21.6 | -5.14 | -10.29; 0.00 | 0.050 |  | 52.7 | 24.5 | -8.10 | -16.81; 0.62 | 0.069 |
| **UCSD-SOBQ** | 41.6 | 31.0 |  | 60.5 | 30.3 | 18.90 | 8.47; 29.34 | <0.001 |  | 65.5 | 33.7 | 23.97 | 5.89; 42.05 | 0.010 |
| **WHO-5** | 14.6 | 5.8 |  | 12.8 | 6.0 | -1.83 | -3.40; -0.26 | 0.023 |  | 12.9 | 5.3 | -1.66 | -3.87; 0.55 | 0.141 |
|  |  |  |  |  |  |  |  |  |  |  |  |  |  |  |
|  |  |  |  |  |  |  |  |  |  |  |  |  |  |  |
| ***Change in QoL between baseline and last follow-up by change DLCO% predicted between baseline and 1-year follow-up*** | | | | | | | | | | | | | | |
|  |  |  |  |  |  |  |  |  |  |  |  |  |  |  |
| **SGRQ** | 0.0 | 17.0 |  | 7.6 | 18.5 | 7.68 | 2.87; 12.49 | 0.002 |  | 13.2 | 18.0 | 13.24 | 5.98; 20.50 | <0.001 |
| **SGRQ symptoms** | -4.0 | 21.6 |  | 1.8 | 22.2 | 5.71 | -0.22; 11.65 | 0.059 |  | 4.4 | 21.8 | 8.34 | -0.58; 17.25 | 0.067 |
| **SGRQ activity** | 3.6 | 22.4 |  | 8.6 | 21.9 | 4.99 | -1.06; 11.03 | 0.105 |  | 13.5 | 16.2 | 9.93 | 2.51; 17.36 | 0.009 |
| **SGRQ impacts** | 0.5 | 17.4 |  | 8.6 | 20.7 | 8.13 | 2.77; 13.48 | 0.003 |  | 16.2 | 18.1 | 15.68 | 7.38; 23.98 | <0.001 |
| **EQ-5D VAS** | 0.4 | 22.5 |  | -9.2 | 23.9 | -9.60 | -15.51; -3.68 | 0.002 |  | -8.9 | 23.5 | -9.32 | -18.23; -0.42 | 0.040 |
| **UCSD-SOBQ** | -0.9 | 30.4 |  | 15.9 | 26.9 | 16.79 | 5.12; 28.46 | 0.005 |  | 18.1 | 25.6 | 18.92 | 2.23; 35.62 | 0.027 |
| **WHO-5** | 0.9 | 5.4 |  | -2.4 | 5.8 | -3.28 | -4.77; -1.78 | <0.001 |  | -1.7 | 5.9 | -2.58 | -4.96; -0.20 | 0.034 |

beta, regression coefficient; CI, confidence interval; EQ-5D VAS, EuroQol five-dimensional questionnaire, recorded as a visual analog scale; SD, standard deviation; SGRQ, St. George’s Respiratory Questionnaire; USCD-SOBQ, University of California San Diego Shortness of Breath Questionnaire; WHO-5, World Health Organization-5 Well-Being Index.

# Table S3: Quality of life scores at visit prior to hospitalisation and subsequent visit after hospitalisation in follow-up

|  |  | **Visit prior to hospitalisation** | | **Visit after hospitalisation** | | |  | |
| --- | --- | --- | --- | --- | --- | --- | --- | --- |
|  | **n** | **mean** | **SD** |  | **mean** | **SD** |  | **p-value** |
| **SGRQ** | 131 | 53.3 | 18.9 |  | 58.8 | 19.7 |  | 0.003 |
| **SGRQ symptoms** | 128 | 61.9 | 21.7 |  | 61.7 | 21.3 |  | 0.556 |
| **SGRQ activity** | 132 | 68.8 | 19.4 |  | 74.1 | 20.7 |  | 0.006 |
| **SGRQ impacts** | 121 | 41.6 | 21.5 |  | 48.2 | 22.4 |  | 0.002 |
| **EQ-5D VAS** | 134 | 57.7 | 17.3 |  | 51.3 | 20.4 |  | 0.001 |
| **UCSD-SOBQ** | 83 | 58.5 | 27.4 |  | 69.3 | 28.3 |  | 0.029 |
| **WHO-5** | 128 | 13.8 | 6.0 |  | 11.9 | 6.1 |  | 0.001 |

EQ-5D VAS, EuroQol five-dimensional questionnaire, recorded as a visual analog scale; SD, standard deviation; SGRQ, St. George’s Respiratory Questionnaire; USCD-SOBQ, University of California San Diego Shortness of Breath Questionnaire; WHO-5, World Health Organization-5 Well-Being Index.
