# Supplementary material for: The clinical course of idiopathic pulmonary fibrosis and its association to quality of life over time: longitudinal data from the INSIGHTS-IPF registry
Source: Respir Res. 2019 Mar 15;20:59. doi: 10.1186/s12931-019-1020-3 (PMC6420774; doi:10.1186/s12931-019-1020-3)

**Additional File 3**

# Figure S1 Estimated mean difference in QoL by the number of hospitalisations compared to patients who were not hospitalized during follow-up


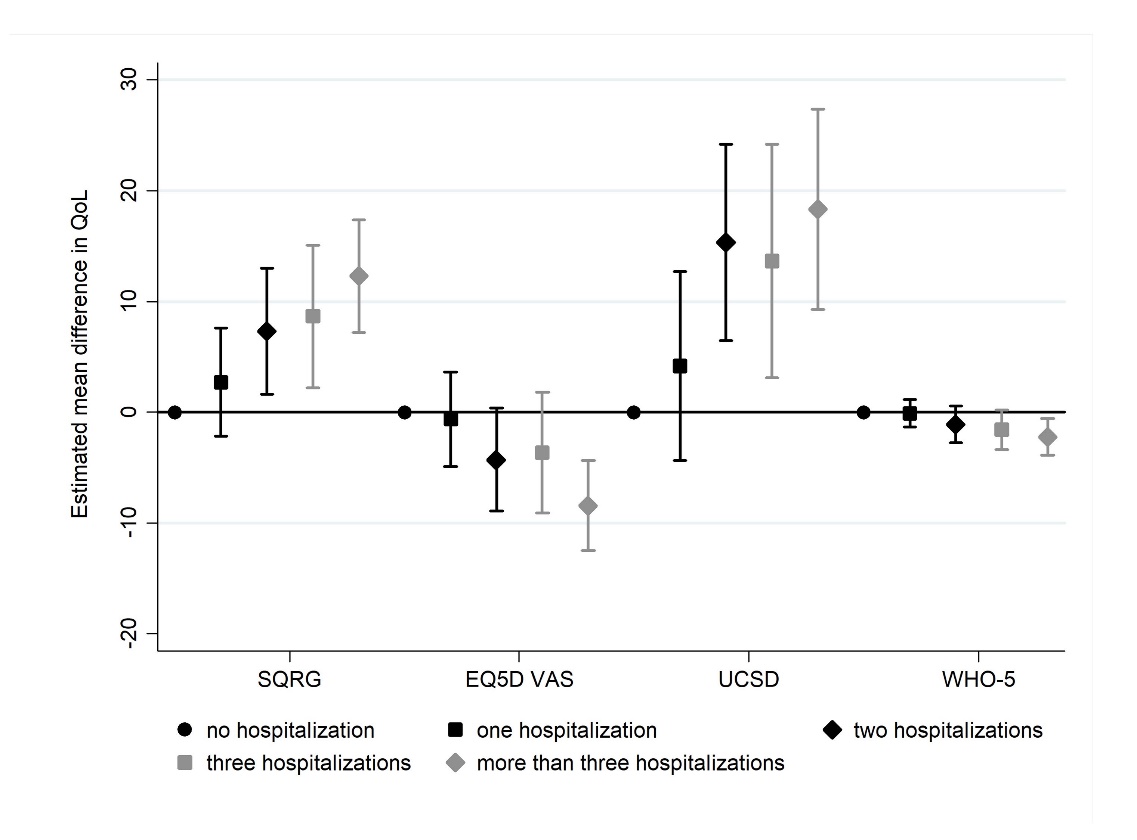

Supplement: Supplementary file 3 — Figure S1. Mean difference in QoL scores for patients with one, two, three and more than three hospitalizations compared to patients who were not hospitalized during the follow-up. (EQ-5D VAS, EuroQol five-dimensional questionnaire, recorded as a visual analog scale; QoL, quality of life; SGRQ, St. George’s Respiratory Questionnaire; USCD-SOBQ, University of California San Diego Shortness of Breath Questionnaire; WHO-5, World Health Organization-5 Well-Being Index). (DOCX 110 kb) [file 12931_2019_1020_MOESM3_ESM.docx]
